# Extracranial Intraluminal Extension of Atypical Meningioma within the Internal Jugular Vein

**DOI:** 10.1155/2013/875607

**Published:** 2013-02-14

**Authors:** Nizar Taki, Richard O. Wein, Harprit Bedi, Carl B. Heilman

**Affiliations:** ^1^Department of Otolaryngology-HNS, Tufts Medical Center, 800 Washington Street, Box 850, Boston, MA 02111, USA; ^2^Department of Radiology, Tufts Medical Center, 800 Washington Street, no. 299, Boston, MA 02111, USA; ^3^Department of Neurosurgery, Tufts Medical Center, 800 Washington Street, no. 178, Boston, MA 02111, USA

## Abstract

Meningiomas represent the most common benign intracranial neoplasms, and may spread by direct extension into nearby venous sinuses, but gross spread to the extracranial venous system is uncommon. We report the case of a patient with extracranial intraluminal spread of meningioma within the internal jugular vein to level III of the neck. Review of the preoperative assessment and management is also presented.

## 1. Introduction 

Meningiomas are the most common benign intracranial neoplasms and are typically well controlled with complete surgical resection. However, atypical meningiomas (WHO grade II) are associated with an increased risk of recurrence and mortality. Extracranial extension of meningioma is seen in less than 2% of cases and usually occurs by direct extension through the skull, perineural spaces, or vascular channels. Though meningiomas may invade dural venous sinuses, extension of the tumor through the sinuses into the extracranial venous system is uncommon [1].

We present a case of extracranial spread of an atypical meningioma extending into the internal jugular vein and its branches. A review of the literature and discussion of extracranial meningiomas are also presented. 

## 2. Case Presentation

**Figure 1:**
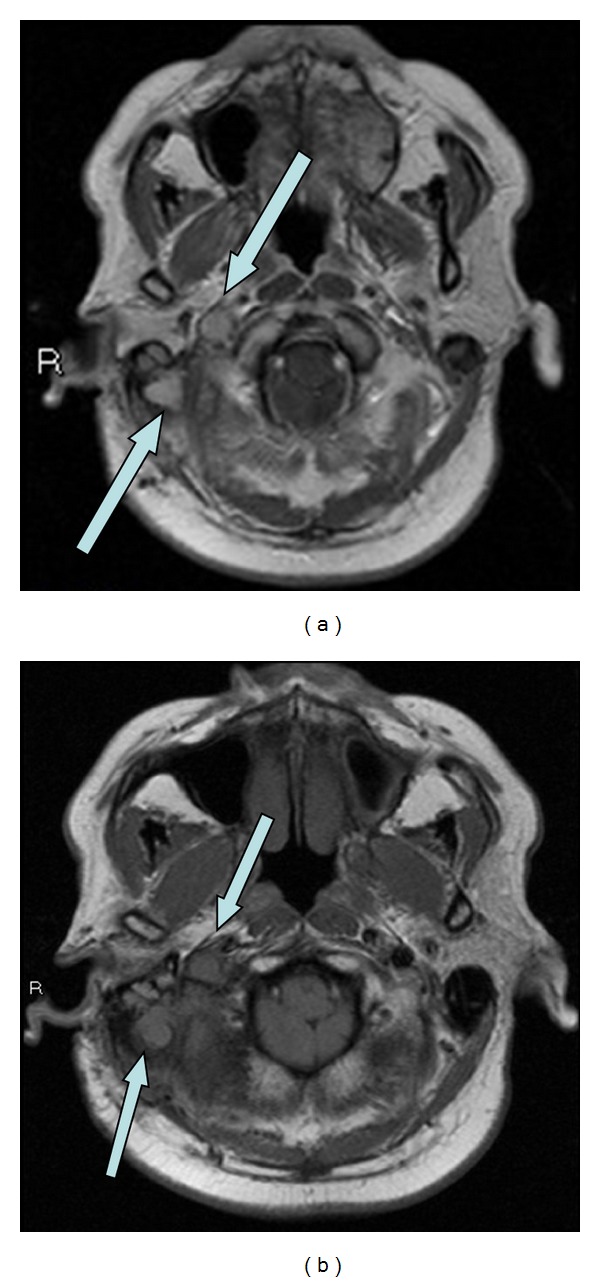
Pre- and postcontrast axial T1 weighted images show an enhancing mass in the region of the right sigmoid plate and sinus. There is an enhancing component involving and enlarging the right internal jugular vein.

**Figure 2:**
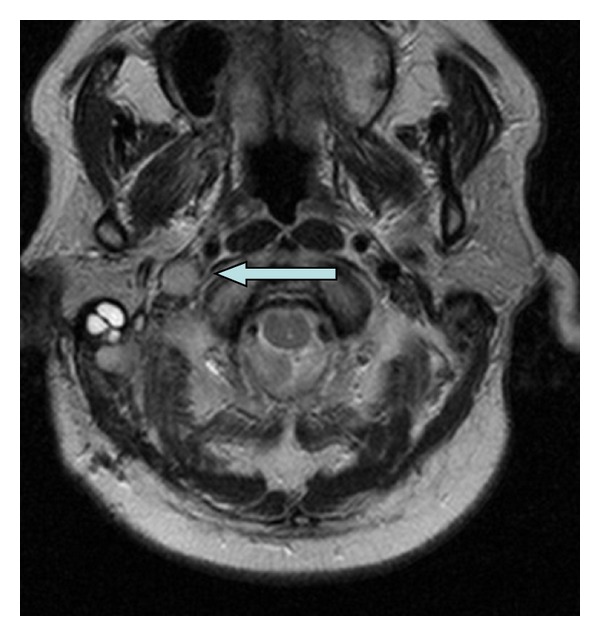
Axial T2 weighted image shows the lack of the normal flow void in the right internal jugular vein secondary to extension of the meningioma. Compare this finding to the normal appearing internal jugular flow void on the left side.

**Figure 3:**
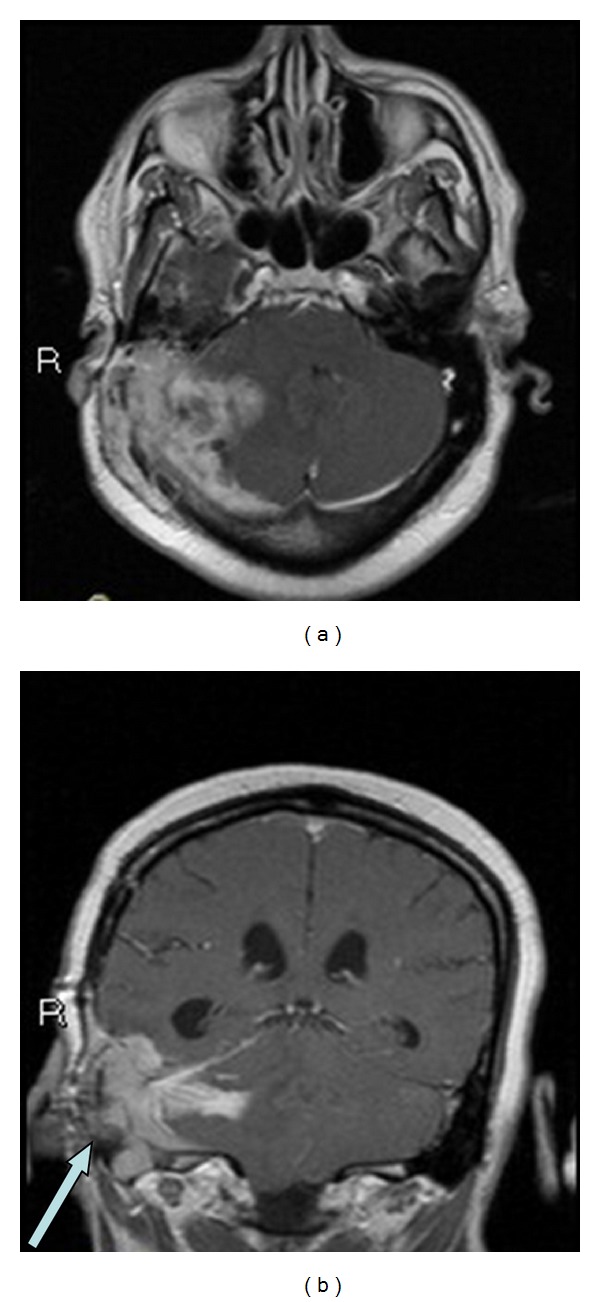
Axial and coronal postcontrast T1 weighted images show the extent of supratentorial and infratentorial enhancement. The coronal image clearly shows the invasion of the right sigmoid sinus (arrow).

A 79-year-old male with a recurrent right posterior fossa meningioma, treated previously with surgical resection, gamma knife radiosurgery, reresection, fractionated radiation therapy, and chemotherapy with temozolomide and bevacizumab over a 7-year period, presented for evaluation with an exophytic postauricular mass. At the time of initial assessment, the patient complained of retroauricular pain, tinnitus, and right-sided hearing loss. On physical exam, a 5 × 6 cm fungating mass was seen at the postauricular crease. Cranial nerves II through XII were grossly intact. CT of the head showed extracranial growth of tumor abutting the posterior and middle cranial fossa with mass effect on the cerebellar hemisphere and the fourth ventricle. MRI of the brain demonstrated the heterogeneous mass to have intracranial and extracranial components involving both the supra- and infratentorial compartments (Figures 1(a), 1(b), 2, 3(a), and 3(b)). The extracranial component extended from the temporalis muscle to the C1-C2 articulation. The lesion measured 6.9 × 6.5 cm at its largest dimension. MRA revealed enhancement in the right middle cranial fossa and the soft tissues overlying the squamous portion of the temporal bone extending to the upper neck adjacent to C2 vertebral body. MRV showed a lack of visualization of the right transverse and sigmoid sinus as well as the internal jugular vein.

**Figure 4:**
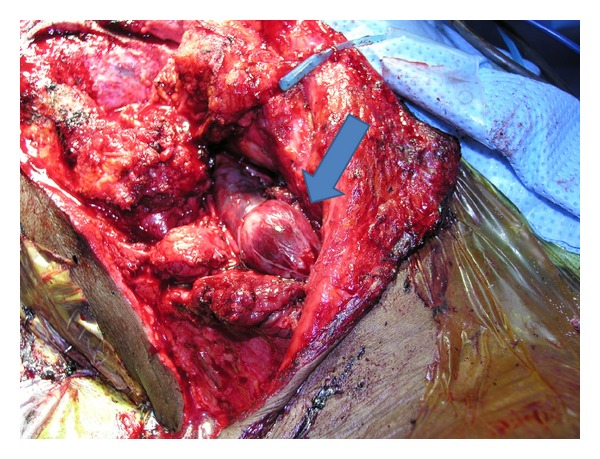
Intraoperative photo demonstrating extension of meningioma within the internal jugular vein to the midneck (arrow).

**Figure 5:**
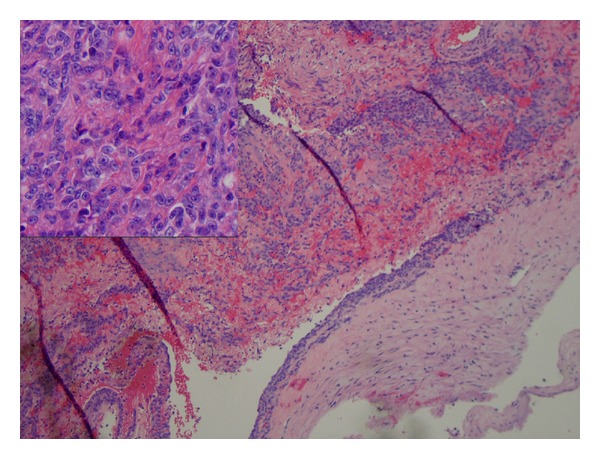
Medium power (100x) H&E stain of internal jugular vein wall with luminal tumor. Inset (400x) demonstrating atypical features of meningioma.

The patient underwent a single-stage surgical resection of the meningioma that involved the temporal bone, suboccipital and jugular foramen, and extracranial components. The resection required removal of the external ear and temporomandibular joint and included mastoidectomy, partial labyrinthectomy, parotidectomy, and modified radical neck dissection with sacrifice of right internal jugular vein (IJV) (Figure 4). Of note, intravascular meningioma was noted to completely fill the IJV extending from the intracranial component to level III of the midneck with a portion branching into the facial vein. Frozen sections confirmed presence of tumor within the vessel lumen (Figure 5). Negative margins were obtained at the time of surgical resection. The final pathology was consistent with WHO grade II atypical meningioma. The surgical defect was closed with using a pectoralis major myocutaneous flap.

## 3. Discussion

Atypical (WHO grade II) meningiomas represent approximately 20–35% of all meningiomas and are associated with an increased risk of recurrence and mortality [2]. Extracranial presentation of meningioma is uncommon and may occur with direct extension, metastasis, or with primary extracranial meningioma. Our case is an example of the first scenario, which is also the most common of the three presentations [3]. 

As intracranial meningiomas grow, they typically displace but do not invade adjacent neural tissues yet may invade bone through haversian canals. The tumor may infiltrate the dura and adjacent venous sinuses, but it is far less likely for the tumor to extend into the extracranial draining vein. Meningiomas involving the jugular foramen are uncommon and can present surgical challenges that may require a staged approach to surgical management. Although the treatment choice is surgery, resection can be associated with compromise of multiple cranial nerves (in particular CN VII and X) and is also associated with an increased local recurrence rate [4]. Although rare, cases in which meningioma has grown within the lumen of the internal jugular vein (without specific endothelial attachment) have been reported. In 3 separate reports, with extracranial intraluminal IJV spread, a 2-stage resection was performed. The initial procedure addressed the supratentorial or intracranial component. The subsequent surgery addressed the management of the extracranial disease [5–7]. Behbahani et al. described a case of atypical meningioma extension to the mediastinum via the internal jugular vein [8]. Schmidt et al. reported a case of a patient presenting with a neck mass that was later diagnosed as having meningioma with direct internal jugular vein extension [9]. Extracranial extension of meningioma at the jugular foramen can mimic the presentations of schwannoma or a glomus jugulare tumor and requires appropriate preoperative assessment for this differential diagnosis [3, 4]. 

 Recurrent locally invasive presentations of meningioma, in particular those involving the jugular foramen, frequently require presurgical evaluation utilizing CT, MRI, and MRA/MRV. CT scan may demonstrate a dural-based lesion with smooth contours and can be useful for identifying hemorrhage or the permeative-sclerotic appearance consistent with bony erosion. On T1 weighted-image (WI) MRI, meningioma is a hypo- to isointense mass with the absence of high-velocity flow voids. In contrast, dense uniform enhancement is noted spreading centrifugally along dural surfaces. On T2 WI, atypical meningiomas may vary being hypo- to hyperintense dependent upon the degree of cellularity associated with the tumor. MRV can be used to confirm patency of adjacent dural sinuses prior to surgery [1].

## 4. Conclusion

Extracranial extension of meningioma is uncommon, and direct intraluminal spread within the internal jugular vein to the neck is a rare finding. Recurrent cases with atypical pathology may represent a scenario with a higher likelihood for this presentation. Appropriate preoperative imaging and treatment planning are essential in these patients.
